# Prognostic stromal gene signatures in breast cancer

**DOI:** 10.1186/s13058-015-0530-2

**Published:** 2015-02-21

**Authors:** Sofia Winslow, Karin Leandersson, Anders Edsjö, Christer Larsson

**Affiliations:** Department of Laboratory Medicine, Translational Cancer Research, Lund University, Medicon Village, Building 404:C3, Lund, 223 81 Sweden; Department of Laboratory Medicine Malmö, Center for Molecular Pathology, Lund University, SUS Malmö, Jan Waldenströms gata 59, Malmö, 205 02 Sweden; Department of Clinical Pathology, University and Regional Laboratories Region Skåne, Jan Waldenströms gata 59, Malmö, 205 02 Sweden; Current affiliation: Department of Biomedicine, Sahlgrenska Cancer Center, University of Gothenburg, Medicinaregatan 1 F, Gothenburg, 405 30 Sweden

## Abstract

**Introduction:**

Global gene expression analysis of tumor samples has been a valuable tool to subgroup tumors and has the potential to be of prognostic and predictive value. However, tumors are heterogeneous, and homogenates will consist of several different cell types. This study was designed to obtain more refined expression data representing different compartments of the tumor.

**Methods:**

Formalin-fixed paraffin-embedded stroma-rich triple-negative breast cancer tumors were laser-microdissected, and RNA was extracted and processed to enable microarray hybridization. Genes enriched in stroma were identified and used to generate signatures by identifying correlating genes in publicly available data sets. The prognostic implications of the signature were analyzed.

**Results:**

Comparison of the expression pattern from stromal and cancer cell compartments from three tumors revealed a number of genes that were essentially specifically expressed in the respective compartments. The stroma-specific genes indicated contribution from fibroblasts, endothelial cells, and immune/inflammatory cells. The gene set was expanded by identifying correlating mRNAs using breast cancer mRNA expression data from The Cancer Genome Atlas. By iterative analyses, 16 gene signatures of highly correlating genes were characterized. Based on the gene composition, they seem to represent different cell types. In multivariate Cox proportional hazard models, two immune/inflammatory signatures had opposing hazard ratios for breast cancer recurrence also after adjusting for clinicopathological variables and molecular subgroup. The signature associated with poor prognosis consisted mainly of *C1Q* genes and the one associated with good prognosis contained *HLA* genes. This association with prognosis was seen for other cancers as well as in other breast cancer data sets.

**Conclusions:**

Our data indicate that the molecular composition of the immune response in a tumor may be a powerful predictor of cancer prognosis.

**Electronic supplementary material:**

The online version of this article (doi:10.1186/s13058-015-0530-2) contains supplementary material, which is available to authorized users.

## Introduction

Gene expression profiling has enabled novel classification of breast cancer into different subgroups. At least five molecular subgroups (basal-like, normal-like, HER2-positive, and luminal A and luminal B) have been identified [1,2] and additional subclasses continue to be suggested [3-7].

Gene expression analyses of tumor samples are generally performed on whole tumor homogenates and thereby represent a pattern that reflects the expression from all cell types present in the tumor. The tumor microenvironment, comprising a large variety of cells, such as fibroblasts, immune cells, and endothelial cells, can constitute a significant part of the tumor and substantially contribute to observed expression patterns. Cells in the microenvironment can influence cancer progression [8-11] and have been shown to predict tumor outcome and therapy response in breast carcinomas [12-14]. One way to obtain cancer cell and stromal compartment-specific expression patterns is to isolate the compartments by laser capture microdissection (LCM).

For routine histological diagnosis of surgically removed tumors, formalin-fixed paraffin-embedded (FFPE) tissue is normally used and thus a wide range of FFPE tumors are available for gene expression analyses. However, fixation and embedding have a detrimental effect on RNA quality, resulting in fragmentation and chemical modifications and making it of less use for expression analyses [15]. In this study, we have established a procedure for global-gene expression analysis using LCM on FFPE triple-negative breast cancers. Isolation of stromal and cancer cell compartments of the tumor with subsequent analysis of the global mRNA expression revealed compartment-specific gene expression. Expanding the stroma-specific gene set by identifying genes with correlating expression levels using tumor data from The Cancer Genome Atlas (TCGA) database gave rise to 16 gene signatures of stromal genes with highly correlating gene expression. Two signatures, consisting of genes related to an immune response, are of particular interest since they are prognostic in multivariate Cox proportional hazard models in several breast cancer data sets and data sets of other cancers.

## Methods

### Tumor material

Tumor specimens, from subjects that had given informed consent, were obtained from Skåne University Hospital, Malmö. Ethical permission has been obtained from the local Research Ethics Committee in Lund (Dnr 2009/658). Information about the tumors was obtained from the pathology reports. All tumors were negative for estrogen and progesterone receptors and had no ERBB2 (HER2) amplification. Tumors with sufficient amount of cells in the stromal compartment to allow conclusive microarray analyses were selected. Two of the tumors used for microarray analysis were high-grade, grade III (3 + 3 + 3 for tubule formation, nuclear pleomorphism, and mitotic count, respectively), and one was low-grade, NHG (Nottingham histological grade) grade I (1 + 3 + 1 according to the NHG grading system). Two of the tumors were invasive ductal carcinomas, whereas one was reported as a medullary carcinoma.

### Tissue preparation

Five consecutive sections (5 μm) of each tumor were prepared on a microtome and mounted onto polyethylene terephtalate (PET) membrane slides (Leica Microsystems, Wetzlar, Germany). When indicated, 30% ethanol was included. Mounted tissue sections were allowed to dry for 30 minutes in room temperature prior to incubation in −20°C for 24 hours for optimal adhesion. To preserve the RNA quality in the archived FFPE tissue specimens, the blocks used for biomarker analysis during the routine prognostic procedure were stored in 4°C.

### Staining

During method development, staining of the tissue sections with either a cresyl violet LCM staining kit (Ambion, part of Thermo Fisher Scientific, Waltham, MA, USA) or standard hematoxylin-and-eosin staining was evaluated. For further analysis, cresyl violet staining was used. Tissue sections were initially rinsed 2 × 1 minute with xylene for deparaffinization and rinsed in 100%, 75%, and 50% ethanol for 30 seconds. Rehydration of the tissue with diethylpyrocarbonate (DEPC)-treated water was performed prior to 40-second incubation in staining solution, followed by additional rinsing with DEPC-treated water for 30 seconds and dehydration with 100% ethanol twice for 30 seconds. Sections were thereafter dried in room temperature and immediately used for LCM or stored at 4°C for up to 1 week.

### Laser capture microdissection

LCM to isolate tumor compartments was performed on a Leica LMD6500. After microdissection, the tissue was collected in 0.5-mL polymerase chain reaction (PCR) tube caps containing Allprep RNA/DNA FFPE kit lysis buffer (Qiagen, Hilden, Germany) with Proteinase K.

### RNA extraction and hybridization

RNA was extracted by using Allprep RNA/DNA FFPE kit (Qiagen) and evaluated with Nanodrop and Bioanalyzer (Agilent Technologies, Santa Clara, CA, USA) in accordance with standard procedures, including calculation of RNA integrity number (RIN) values as previously described [16]. Isolated RNA samples with a 260/280 ratio of at least 1.8 and RIN value of more than 2.0 were used for amplification with SensationPlus FFPE Amplification and WT labeling kit (Affymetrix, Santa Clara, CA, USA) in accordance with the protocol of the supplier. The resulting ds-cDNA was hybridized to a Human Gene 1.0 ST array (Affymetrix).

### Data sets

TCGA expression data were downloaded November 2013 (breast cancer), January 2014 (kidney and lung cancer), and March 2014 (head and neck cancer) from the TCGA database [17]. The data were log2-transformed after the addition of 1 to each normalized value. Clinical and follow-up data were downloaded in May 2014. The basic patient characteristics of the TCGA breast cancer data used for follow-up analyses can be found in Additional file 1: Table S1. The NKI295 [18], Wang [19], and TransBig [20] datasets were downloaded in an assembled form, as described [21]. The original array data can be found at [22] (NKI295) or on the National Center for Biotechnology Information Gene Expression Omnibus website as GSE2034 and GSE7390.

### Data analysis

All analyses were done with R by using the limma, survival, and cluster packages. Breast cancer subtypes were determined by using the nearest correlations with the PAM50 centroids [23]. For analyses of gene signatures from the Wang and TransBig data sets, probes with a mean log2 signal of more than 6 were included, and for the NKI295 dataset a threshold of −0.3 was used.

## Results

### Isolation of RNA from laser capture-microdissected FFPE breast tumors

To obtain RNA of sufficient quality from tumor compartments isolated with LCM, sectioning, mounting, deparaffinization, and staining steps were performed with the highest possible purity to avoid RNase contamination. Adhesion of the tissue section to the membrane slide was obtained with 30% ice-cold ethanol (Additional file 2: Table S2) to avoid further degradation. As the standard histological staining with hematoxylin and eosin may influence the RNA integrity negatively [24], cresyl violet was used for tissue staining. We obtained similar RNA quality and amount from a cresyl violet-stained section as from a non-stained section either with or without rehydration (Additional file 3: Table S3 and Additional file 4: Figure S1). Since cresyl violet staining with rehydration was the only procedure that resulted in both an acceptable RNA quality and adequate tissue morphology, this was henceforth used as staining procedure.

**Figure 1:**
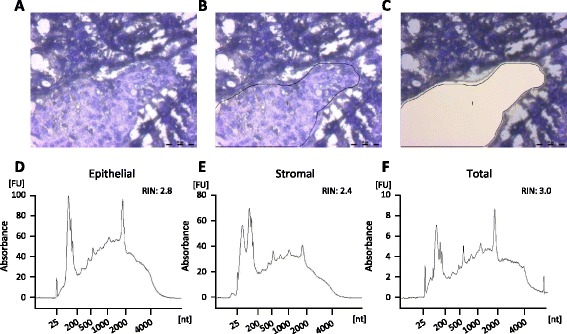
**RNA integrity from laser capture-microdissected malignant epitheluim and surrounding stromal in breast cancer tumor sections.** Cresyl violet staining of a representative triple-negative breast tumor section **(A)** with defined **(B)** and dissected **(C)** cancer cells. Electropherogram depicting RNA from microdissected cancer **(D)** and stromal breast tumor **(E)** cells in comparison with RNA from total tissue section **(F)**. RNA length is indicated on the x-axis, and the y-axis corresponds to the fluorescence units (FU). RNA integrity number (RIN) values are calculated according to a standardized algorithm by using various features correlated to RNA integrity. nt, nucleotides.

To collect epithelial and stromal compartments from breast tumor tissue, five consecutive tissue sections from triple-negative tumors were laser-microdissected (Figure 1A-C). We found that collection of 25 to 28 mm^2^ from the cancer cell compartment yielded a sufficient amount of RNA (Table 1A). However, isolation of stromal compartments with or without inflammation showed that stroma with few inflammatory cells did not yield enough material for downstream applications and limited us to analyze only tumors with inflammatory stroma. Analysis of three triple-negative tumors with inflammatory stroma showed a similar relationship between dissected tissue area and extracted RNA amount for both compartments (Table 1B-D and Figure 1D-F).

**Table 1:** **Collected RNA and RNA integrity number values of laser-microdissected breast tumor cells using Bioanalyzer and Nanodrop analysis**

		**Extracted RNA**			
	**Isolated tissue area**	**Bioanalyzer**		**Nanodrop**	
	**mm** ^**2**^	**ng/μL**	**RIN**	**ng/μL**	**260/280**
A)					
Malignant epithelial cells	28	16.8	2.6	44.8	2.1
Benign epithelial cells	25	11.1	2.7	24.7	2.24
Stroma cells (including inflammatory cells)	18.2	10.2	2.3	16.2	2.26
Stroma cells	18.4	0.4	N/A	2.5	−26
Total tissue section	~200	200	2.3	282	2.07
B)					
Epithelial cells	32.65	57.0	2.8	23.31	2.06
Stroma cells	26.5	27.0	2.4	15.59	1.97
Total tissue section	~200	266	3.0	230.5	2.05
C)					
Epithelial cells	19.25	27.7	2.6	30.62	2.06
Stroma cells	92.35	10.5	2.1	25.33	1.89
Total tissue section	~200	28.8	2.3	48.53	1.59
D)					
Epithelial cells	10.2	1.3	1.2	10.14	1.94
Stroma cells	15.0	7.8	2.1	21.77	1.63
Total tissue section	~200	5.6	2.2	14.51	1.54

RIN, RNA integrity number.

### Identification of stroma- and epithelium-specific gene expression

Extracted and amplified samples were hybridized to an Affymetrix Human Gene ST 1.0 array. To identify genes that are selectively expressed in either the stromal or the cancer cell compartment, the probe list was shrunk by removing probes for which every sample had a log2 expression value below a threshold (less than 7) and by applying a variance filter, which removed probes with variance of less than 0.15. The remaining 5,971 probes were analyzed for difference in expression between stroma and cancer cells by using the limma package in R.

Even if only three tumors were analyzed, this approach identified 107 probes (Additional file 5: Table S4), with an adjusted *P* value of less than 0.05, that had higher levels in stroma and 48 probes (Additional file 6: Table S5) that were higher in cancer cells with the same adjusted *P* value (Additional file 7: Figure S2A-B). Several of the genes enriched in the stroma compartment are genes encoding matrix proteins, such as collagens (for example, *COL1A1*) and decorin (*DCN*). There was also enrichment of genes related to an immune response such as chemokines (*CXCL12* and *CCL19*) along with their receptors (*CXCR4* and *CCR7*), matrix metalloproteases (*MMP2* and *MMP9*), the T cell-specific genes *CD4* and granzyme K (*GZMK*), and the B cell-specific immunoglobulin genes such as both heavy (*IGHA1*, *IGHA2*, *IGHV1-5*, and *IGHM*) and light (*IGLJ3*, *IGLV6-57*, *IGKC*, *IGKV1-5*, and *IGK@*) chain-encoding genes. This indicates that the analysis has worked technically and that it identifies genes that are essentially stroma-specific in their expression. On the other hand, several of the genes in the cancer cell compartment are epithelial, such as the cadherin family member desmoglein (*DSG2*), intracellular junction protein desmoplakin (*DSP*), the epithelium-specific transcription factors (*ELF3* and *5*), keratin 7 (*KRT7*), claudin 4 and 7 (*CLDN4* and *7*), and integrin beta 8 (*ITGB8*).

### Stroma-specific gene sets

The genes highly expressed in stroma most likely represent contributions from several different cell types that may be at different maturation stages that are located mainly in the stromal compartment. Therefore, the expression levels of these genes in a tumor homogenate may potentially reflect the combination of these cell types in the tumor. This raises the possibility to identify gene signatures that can be used as an estimate of the molecular and of the cellular composition of the stroma in a tumor. However, the method we have used, LCM of FFPE material followed by amplification and hybridization, is not optimal for an accurate estimate of RNA levels and will conceivably have low sensitivity. Therefore, to expand the set of genes that may constitute specific stromal signatures, we used TCGA mRNA data from 982 primary breast cancers and identified all genes whose expression level correlated with a correlation coefficient above 0.85 with at least one of the original genes identified as enriched in the stromal compartment of the tumors in our analysis. This set was further expanded by including genes that had a correlation coefficient above 0.89 with one of the genes in the expanded set. This led to an enlarged set comprising 361 genes. All of these genes were also found to be expressed at higher levels in the stroma in the laser capture-microdissected samples (Additional file 7: Figure S2C). To define signatures of highly correlating genes, an iterative correlation analysis was performed yielding clusters of genes, in which all genes in a cluster had an average correlation coefficient above 0.90 with all other genes in the set. Owing to the large amount of genes typical of an immune or an inflammatory response, the threshold was set to 0.91 for these genes. This step yielded 16 gene signatures which contained at least three genes (Table 2 and Additional file 7: Figure S2C).

**Table 2:** **Stromal gene signatures of highly correlating genes**

**1**	**2**	**3**	**4**	**5**	**6**		**7**	**8**
*COL1A2*	*DCN*	*GIMAP5*	*CLEC14A*	*PTPRB*	*CD48*	*SLAMF1*	*BTK*	*PARVG*
*COL3A1*	*GLT8D2*	*GIMAP4*	*CXorf36*	*TEK*	*PTPN7*	*TBX21*	*NCKAP1L*	*NCF4*
*COL5A2*	*LUM*	*GIMAP7*	*ARHGEF15*	*ELTD1*	*IL2RG*	*CD96*	*DOCK2*	*FERMT3*
*FAP*		*GIMAP6*	*CD34*		*ACAP1*	*GZMA*	*CD4*	*WAS*
*COL1A1*		*GIMAP8*	*TIE1*		*CD247*	*GZMK*	*FYB*	*CYTH4*
*COL5A1*			*ESAM*		*CD27*	*ITK*	*PLEK*	*MYO1F*
*COL6A3*			*CDH5*		*CD2*	*PYHIN1*	*KLHL6*	*HCLS1*
*ADAM12*			*ROBO4*		*CD3D*	*SAMD3*	*EVI2B*	*ARHGAP9*
*DACT1*			*MYCT1*		*CD3E*	*SCML4*	*LCP2*	*CD37*
*FBN1*					*CD5*	*SH2D1A*	*CD53*	
*POSTN*					*CXCR3*	*SLAMF6*	*PTPRC*	
*THBS2*					*LY9*	*TRAT1*	*IL10RA*	
*CDH11*					*SIT1*	*ZNF831*	*SPN*	
*VCAN*					*SLA2*	*TIGIT*	*SNX20*	
					*UBASH3A*	*CD3G*	*CCR5*	
					*LCK*	*CXCR6*	*SASH3*	
					*SIRPG*		*IKZF1*	
9	10	11	12	13	14	15	16	
*TBC1D10C*	*SLC7A7*	*CCL5*	*HLA-DOA*	*C1QC*	*FCRL5*	*PTPRCAP*	*LST1*	
*TMC8*	*CD86*	*NKG7*	*HLA-DPA1*	*C1QB*	*POU2AF1*	*S1PR4*	*AIF1*	
*RASAL3*	*LILRB1*	*PRF1*	*HLA-DPB1*	*C1QA*	*CD79A*	*GZMM*	*TNFAIP8L2*	
*CORO1A*	*LAIR1*		*HLA-DRA*	*TYROBP*	*TNFRSF17*	*ZAP70*		
	*LAPTM5*		*HLA-DMB*	*SPI1*	*LOC96610*			
					*ADAM6*			

**Figure 2:**
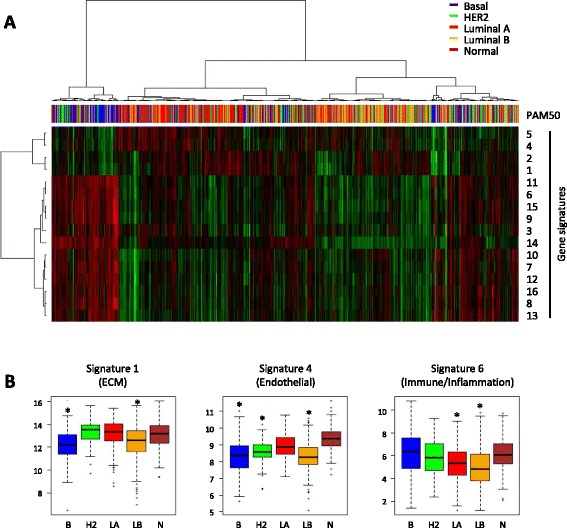
**Expression levels of gene signatures in breast cancer tumors. (A)** The means of the log2 expression levels of the genes in each signature were calculated for 982 breast cancers by using data from The Cancer Genome Atlas project. The tumors were subjected to non-supervised hierarchical clustering based on the signatures. Subtypes, determined by PAM50 centroids, are indicated in color. **(B)** The mean log2 expression levels of the genes in the signatures with the largest number of genes representing (ECM) genes (signature 1), endothelial genes (signature 4), and immune/inflammatory genes (signature 6) are shown for the indicated breast cancer subgroups. Asterisk indicates that the value is lower than in the groups without asterisk; *P* <10^−7^ (analysis of variance followed by Tukey’s honest significance test) except signature 4 (HER2 versus luminal A, *P* = 0.0016) and for signature 6 (luminal A versus HER2, *P* = 0.030; luminal B versus HER2, *P* = 0.000018; and luminal B versus normal, *P* = 0.0000062).

For each signature, an aggregated value was calculated for each tumor by taking the arithmetic mean of the log2 expression of the genes in the set. The breast cancer tumors in the TCGA database were thereafter clustered by using the signature scores as variables (Figure 2A). There was no obvious relationship between this clustering and the breast cancer molecular subtype, determined by the PAM50 centroids. However, the gene signatures were separated into three major groups: one with primarily matrix/fibroblast-related genes (signatures 1 and 2), one with endothelium-associated genes (signatures 4 and 5), and one with genes typical for immune/inflammatory cells. We thereafter compared the aggregated expression levels for the signatures in the molecular subtypes (Figure 2B demonstrates the signature with the largest number of genes, all signatures are shown in Additional file 8: Figure S3). Luminal B tumors were low in expression of all stromal signatures, whereas basal-like tumors were low in matrix/fibroblast and endothelial genes but high in immune/inflammatory signatures. HER2-enriched tumors were low in endothelial genes and luminal A in immune/inflammatory signatures. Thus, the tumor group associated with good prognosis (luminal A) was high in both matrix and endothelial genes.

### Prognostic value of gene signatures

To assess whether the identified signatures of highly correlated genes may have prognostic implications, univariate Cox proportional hazard regression analyses were performed for the tumors from the TCGA database that had follow-up data (Table 3). The standardized mean of log2 expression levels of all the genes in a set were computed and used for the analyses. None of the gene signatures, except gene set 5, which is a signature with endothelial-related genes, was associated with risk of recurrence. The hazard ratio (HR) of signature 5 was 0.714.

**Table 3:** **Univariate Cox proportional hazard analysis of breast cancers using standardized mean values for the gene sets as variables and new tumor event as end point**

	**HR**	**95% CI**	***P*** **value**
Gene signature 1	1.13	0.821-1.55	0.454
Gene signature 2	1.03	0.759-1,41	0.835
Gene signature 3	0.776	0.578-1.04	0.0922
Gene signature 4	0.816	0.615-1.08	0.157
Gene signature 5	0.714	0.547-0.932	0.0132
Gene signature 6	0.852	0.630-1.15	0.297
Gene signature 7	0.951	0.706-1.28	0.742
Gene signature 8	1.05	0.784-1.42	0.728
Gene signature 9	0.956	0.708-1.29	0.768
Gene signature 10	1.13	0.834-1.54	0.426
Gene signature 11	0.898	0.659-1.22	0.495
Gene signature 12	0.852	0.639-1.14	0.276
Gene signature 13	1.18	0.874-1.59	0.283
Gene signature 14	0.864	0.632-1.18	0.360
Gene signature 15	0.888	0.653-1.21	0.447
Gene signature 16	1.04	0.768-1.40	0.806

CI, confidence interval; HR, hazard ratio.

In a full multivariate model, using all gene sets, some signatures had *P* values of less than 0.05 (not shown), indicating that a multivariate model may be more appropriate. Therefore, we performed both forwards and backwards selection to identify an optimal model. The *P* value of the likelihood ratio test of the model was used as an indicator of the quality of the model. This resulted in an optimal model that contained four signatures. A multivariate analysis with these signatures indicated that two (1 and 13) were associated with an increased and two (5 and 12) with a decreased risk for new tumor event (Table 4A). To analyze whether the HRs may be due to confounding effects of other parameters, we included tumor size, node stage, and estrogen receptor status in the model and stratified for stage (Table 4B). We also analyzed a model in which stratification was done for PAM50 subtype (Table 4C). This showed that the gene sets, as well as node stage and estrogen receptor status, are independent prognostic markers. The only exception was signature 5, which had a *P* value of more than 0.05 upon stratification for PAM50 subtype.

**Table 4:** **Mulivariate Cox proportional hazard model using standardized mean values for the gene signatures as variables**

	**HR**	**95% CI**	***P*** **value**
A) Multivariate model, not stratified			
Gene signature 1	1.790	1.205-2.659	0.00392
Gene signature 5	0.6210	0.4201-0.9177	0.0168
Gene signature 12	0.3085	0.1592-0.5977	0.000492
Gene signature 13	3.327	1.743-6.353	0.000270
B) Multivariate model stratified for stage			
Gene signature 1	1.870	1.20-2.91	0.00535
Gene signature 5	0.5847	0.371-0.922	0.0209
Gene signature 12	0.2685	0.115-0.630	0.00250
Gene signature 13	3.238	1.47-7.13	0.00353
Tumor size	1.158	0.384-3.49	0.794
Node status	3.603	1.33-9.74	0.0115
Estrogen receptor	2.928	1.40-6.14	0.00446
C) Multivariate model stratified for PAM50 subtype			
Gene signature 1	2.099	1.35-3.27	0.00103
Gene signature 5	0.6650	0.437-1.01	0.0569
Gene signature 12	0.3118	0.147-0.662	0.00242
Gene signature 13	3.026	1.52-6.01	0.00158
Node status	4.578	2.07-10.1	0.000175
D) Multivariate model, not stratified			
Gene signature 12	0.3016	0.1686-0.5394	0.0000532
Gene signature 13	3.406	1.860-6.235	0.0000713
E) Multivariate model stratified for stage			
Gene signature 12	0.2573	0.128-0.518	0.000140
Gene signature 13	3.451	1.77-6.73	0.000278
Tumor size	0.9399	0.311-2.84	0.913
Node status	3.905	1.43-10.7	0.00785
Estrogen receptor	2.842	1.37-5.89	0.00498
F) Multivariate model stratified for PAM50 subtype			
Gene signature 12	0.3576	0.1873-0.6827	0.00183
Gene signature 13	2.759	1.470-5.179	0.00159
Node status	4.533	2.563-9.994	0.000179

In B and E, variables included tumor size (>20 versus ≤20 mm), node status (positive versus negative), and estrogen receptor status (negative versus positive) and the model was stratified for stage. In C and F, the model was stratified for PAM50 subtype. CI, confidence interval; HR, hazard ratio.

Gene signatures 12 and 13 both have an inflammatory/immune profile with set 12 containing *HLA* genes and set 13 *C1Q* genes. These were the most influential signatures in the multivariate Cox model and their opposing HRs indicate that the characteristic of the immune response in a tumor has prognostic value. Multivariate analyses with only these sets demonstrated significant HRs for both sets in models both without or with adjustments for the clinicopathological parameters and PAM50 subtype (Table 4D-F).

### Stromal gene signatures in other tumors

**Figure 3:**
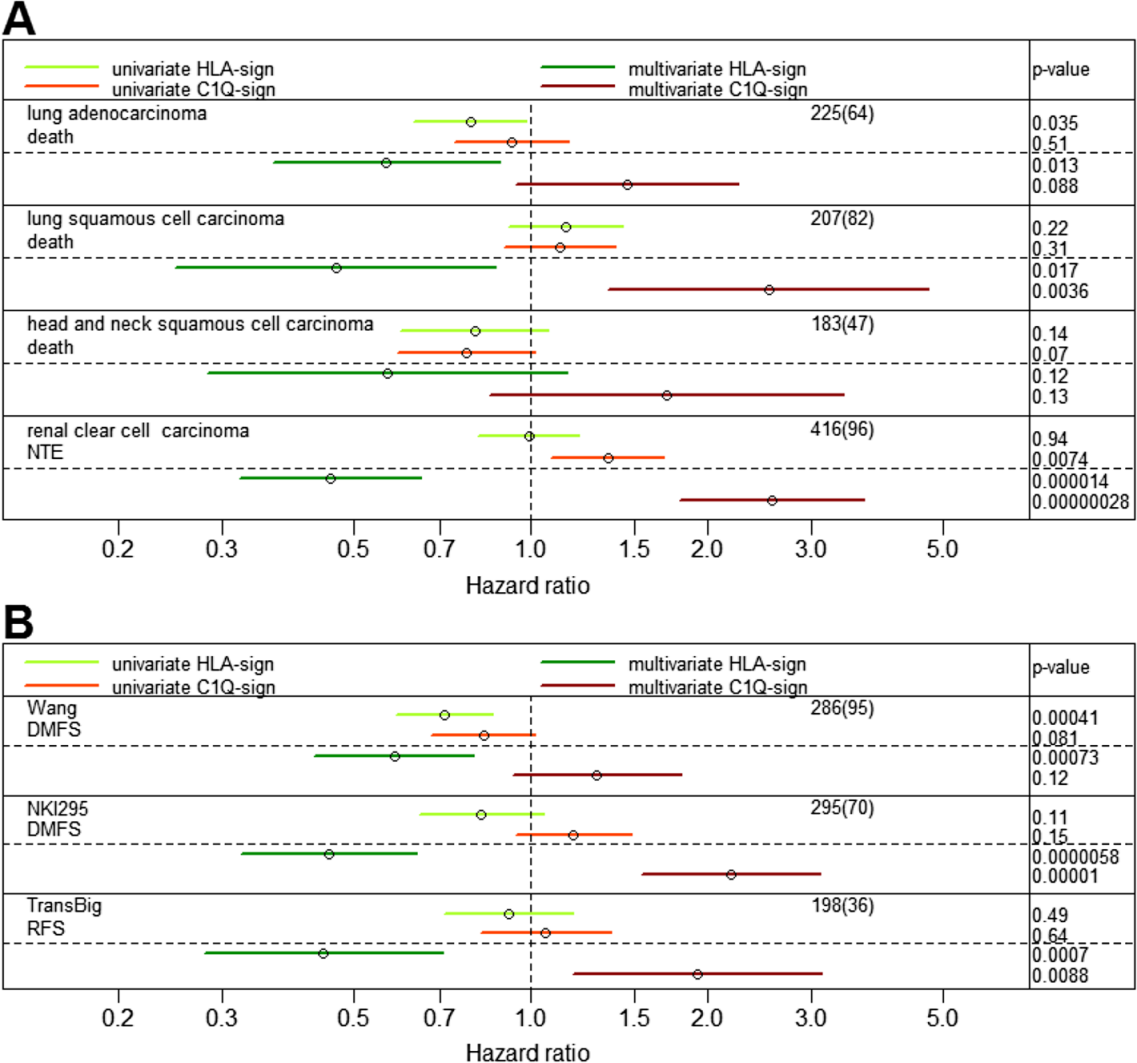
**Hazard ratio of the C1Q and HLA signatures in different tumor forms and other breast cancer data sets.** Lines represent confidence intervals (95%) from uni- and multivariate Cox proportional hazard analyses using the C1Q and HLA gene signatures as variables of different tumors using The Cancer Genome Atlas data **(A)** or three other breast cancer data sets **(B)**. End points are death, new tumor event (NTE), distant metastasis-free survival (DMFS), or recurrence-free survival (RFS). The numbers of cases and events for each data set, within parentheses, are shown for each data set.

The analyses indicate that the profile of an immune/inflammatory response in the tumor has a prognostic value. To analyze whether this is general and also applies to other tumors, we used RNA HiSeq data from four other cancer forms available in the TCGA database (Figure 3A). For both renal clear cell carcinoma, with new tumor event as the end point, and lung squamous cell carcinoma, with death as the end point, a similar pattern with no significant roles of the gene signatures as isolated variables, but with opposing HRs in a multivariate model, was detected. A similar but not significant tendency was seen for lung adenocarcinoma and head and neck squamous cell carcinoma.

We also analyzed the signatures in three other breast cancer data sets, in which the expression had been analyzed with microarray technology (Figure 3B). Here, a threshold of mean log2 expression level for the probes was applied, excluding a few genes from calculation of the gene signature values. For two of the three data sets (NKI295 [18] and TransBig [20]) analyzed, significant associations with opposing HRs was seen in analogy with the breast cancer TCGA data. In the third set (Wang [19]), a similar but, for the C1Q signature, not significant association was observed.

### An immune/inflammatory score

**Figure 4:**
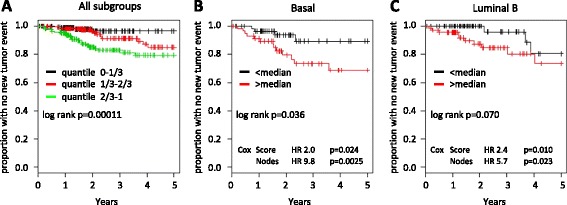
**Kaplan-Meier curves based on the C1Q-HLA score.** A score was calculated as the mean value of the C1Q signature minus the mean value of the HLA signature for each of 504 tumors with survival data from The Cancer Genome Atlas database. **(A)** All breast cancers were grouped in three quantiles of the C1Q-HLA score, and Kaplan-Meier curves were generated. Basal-like **(B)** and luminal B **(C)** breast cancers were grouped on the basis of the C1Q-HLA score above or below the median. Cox indicates the hazard ratio (HR) and *P* values obtained when the C1Q-HLA score was used as a continuous variable together with lymph node status in a multivariate Cox proportional hazard model.

The observed prognostic values of the C1Q and HLA signatures raises the possibility that a prognostic score, based on the ratio of the two signatures, could be calculated. Therefore, we defined a C1Q-HLA score as the difference in mean log2 expression of the two signatures. When Kaplan-Meier curves for the TCGA tumors were constructed on the basis of quantiles of this score, it is evident that the top 1/3 is clearly at higher risk than the bottom 1/3 for recurrence. In the latter group, there were hardly any new tumor events within five years after the initial treatment (Figure 4A).

We also evaluated the molecular subgroups. For HER2-enriched, luminal A, and normal-like tumors, the number of events were too few for relevant analysis, but for basal-like and luminal B tumors, Kaplan-Meier curves based on the C1Q-HLA score above or below the median were constructed (Figure 4B and C). We also evaluated the C1Q-HLA score together with lymph node status in a multivariate Cox proportional hazard model using the basal and luminal B tumors (HR and *P* values are shown in Figure 4). All of these analyses gave *P* values below 0.05 for the score as prognostic indicator, except the log-rank test in luminal B tumors, in which the *P* value was 0.070. Taken together, the analyses indicate that a C1Q-HLA score is of prognostic value also in these isolated subgroups.

### Other immune/inflammatory genes

**Figure 5:**
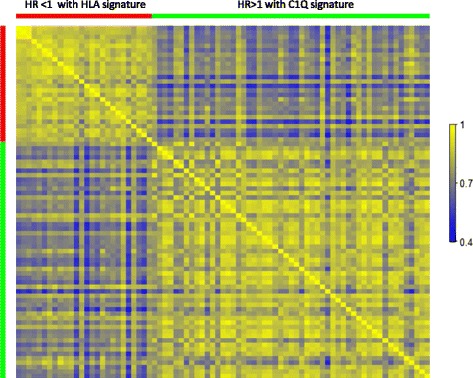
**Correlation matrix of expression levels of genes found to provide altered hazard together with either the C1Q or the HLA signatures.** The correlation coefficients were calculated by using the log2 expression levels of the genes that were found to be associated with either a higher (red bar) or a lower (green bar) hazard in a multivariate Cox analysis together with the HLA or C1Q signature, respectively. The Cancer Genome Atlas breast cancer data were used for the analysis. The genes are listed in Tables 5 and 6. HR, hazard ratio.

The opposing HRs obtained with the HLA and C1Q signatures in several cancer data sets indicate that the profile of the inflammatory components in a tumor has prognostic importance. To screen for other inflammatory genes that may provide prognostic information, we analyzed all immune/inflammation-related genes obtained in the expanded stromal gene set pairwise with both the C1Q and the HLA signatures in multivariate Cox proportional hazard models (Tables 5 and 6). Using a cutoff of 0.01 for the *P* value of the gene in the Cox models resulted in 26 genes with an HR of more than 1 in a multivariate model with the HLA signature and 53 genes with an HR of less than 1 in a model with the C1Q set. To further analyze these genes, a correlation matrix was generated by using the expression data from TCGA tumors (Figure 5). A pattern can be discerned: genes that are associated with higher risk in general are more correlated with each other than with genes that are associated with lower risk, and vice versa.

**Table 5:** **Hazard ratios and** ***P*** **value**s **for indicated genes obtained in multivariate Cox proportional hazard models together with the HLA signature**

**Gene**	**HLA signature (gene set 12)**		**Gene of interest**	
	**HR**	***P*** **value**	**HR**	***P*** **value**
*C1QC*	0.329	0.0000279	3.18	0.0000202
*C1QB*	0.336	0.000103	3.00	0.000114
*C1QA*	0.401	0.000285	2.53	0.000412
*ITGB2*	0.455	0.000206	2.26	0.000426
*LAIR1*	0.352	0.000324	2.86	0.000526
*SPI1*	0.419	0.000374	2.43	0.000571
*FERMT3*	0.423	0.000306	2.45	0.000676
*LILRB1*	0.378	0.000439	2.71	0.000744
*TNFAIP8L2*	0.461	0.000403	2.21	0.000873
*CYTH4*	0.389	0.000401	2.63	0.000967
*MYO1F*	0.344	0.000611	2.92	0.00134
*LRRC25*	0.435	0.000809	2.43	0.00135
*DOK2*	0.421	0.000708	2.45	0.00150
*CD68*	0.625	0.00450	1.67	0.00230
*LAPTM5*	0.451	0.00120	2.25	0.00232
*SLC7A7*	0.457	0.00137	2.25	0.00265
*NCF4*	0.433	0.00152	2.28	0.00276
*LILRB2*	0.481	0.00217	2.12	0.00373
*MS4A6A*	0.311	0.00220	3.04	0.00414
*CD86*	0.450	0.00250	2.23	0.00527
*CD53*	0.386	0.00270	2.49	0.00623
*C3AR1*	0.469	0.00490	2.12	0.00778
*FAM78A*	0.486	0.00366	2.02	0.00813
*LILRB4*	0.510	0.00483	1.91	0.00862
*FCER1G*	0.508	0.00552	1.96	0.00880
*FGD2*	0.565	0.00383	1.74	0.00923

HR, hazard ratio.

**Table 6:** **Hazard ratios and** ***P*** **values for indicated genes obtained in multivariate Cox proportional hazard models together with the C1Q signature**

**Gene**	**C1Q signature (gene set 13)**		**Gene of interest**	
	**HR**	***P*** **value**	**HR**	***P*** **value**
*HLA-DPA1*	3.44	0.00000869	0.289	0.00000287
*GZMH*	2.73	0.0000271	0.332	0.00000330
*GZMA*	2.55	0.0000673	0.369	0.0000203
*GZMK*	2.19	0.000210	0.419	0.0000234
*CD74*	4.33	0.0000469	0.229	0.0000469
*HLA-DPB1*	3.07	0.0000821	0.326	0.0000752
*THEMIS*	2.05	0.000598	0.429	0.000129
*HLA-DRA*	3.31	0.000176	0.311	0.000131
*KIAA0748*	2.12	0.000601	0.439	0.000171
*ITK*	2.15	0.000603	0.443	0.000236
*FGL2*	2.25	0.000732	0.435	0.000342
*SCML4*	1.98	0.00113	0.471	0.000357
*HLA-DMA*	3.15	0.000406	0.318	0.000364
*SLA2*	2.32	0.000697	0.424	0.000556
*CD3D*	2.14	0.00129	0.470	0.000860
*SAMD3*	1.98	0.00164	0.491	0.000870
*HLA-DMB*	3.00	0.000690	0.345	0.000938
*SPN*	2.32	0.000923	0.428	0.00101
*CD8A*	1.91	0.00285	0.513	0.00115
*GPR171*	1.94	0.00228	0.485	0.00125
*CRTAM*	2.18	0.00104	0.441	0.00131
*CD96*	2.07	0.00184	0.474	0.00139
*SH2D1A*	1.93	0.00238	0.504	0.00165
*FASLG*	1.99	0.00206	0.491	0.00172
*RASAL3*	2.53	0.00113	0.393	0.00176
*CCL5*	2.09	0.00244	0.479	0.00195
*TRAT1*	1.81	0.00377	0.524	0.00218
*KLRK1*	1.90	0.00358	0.514	0.00219
*HCST*	2.85	0.00158	0.366	0.00224
*CD2*	2.07	0.00232	0.486	0.00236
*PRKCB*	1.94	0.00321	0.504	0.00280
*ZNF831*	1.76	0.00457	0.535	0.00284
*LCP2*	2.40	0.00216	0.438	0.00293
*KCNA3*	1.67	0.00747	0.559	0.00318
*PTPN7*	2.59	0.00187	0.395	0.00319
*CD52*	1.99	0.00297	0.495	0.00331
*CXCR6*	1.99	0.00376	0.512	0.00342
*GZMM*	1.88	0.00395	0.516	0.00344
*PSTPIP1*	2.40	0.00293	0.441	0.00376
*CCR5*	2.26	0.00315	0.460	0.00394
*CD3E*	2.00	0.00415	0.511	0.00423
*IL12RB1*	2.64	0.00256	0.399	0.00443
*PYHIN1*	1.85	0.00526	0.534	0.00461
*SLAMF6*	1.88	0.00484	0.533	0.00468
*HLA-DOA*	2.02	0.00442	0.505	0.00511
*CD3G*	1.76	0.00627	0.553	0.00559
*IKZF1*	2.02	0.00482	0.491	0.00703
*CD27*	1.75	0.00737	0.570	0.00834
*GMFG*	2.34	0.00472	0.434	0.00868
*LST1*	2.81	0.00446	0.377	0.00868
*TBC1D10C*	2.05	0.00665	0.504	0.00967
*C16orf54*	1.92	0.00683	0.507	0.00993
*CD247*	1.92	0.00776	0.536	0.00998

HR, hazard ratio.

## Discussion

The tumor stroma is composed of several non-malignant cell types that together build up the tumor microenvironment which may promote both tumor progression and metastasis [8,10,11,25]. In this study, we have used LCM for isolation of epithelial and stromal compartments of FFPE triple-negative breast tumors to enable analysis of compartment-specific gene expression. The usage of FFPE tumors is advantageous because of the large amount of routinely stored tumors, but gene expression analysis of this material has been challenging because of chemical modifications and degradation of the RNA [26].

Methodology development has improved the possibilities of accurate analysis of whole transcript expression from FFPE material. Studies thus far have mainly been technically oriented [27,28], but also tumor grade-, prognostic-, and subtype-specific expression for identification of novel gene expression profiles has been analyzed [29-33].

In this study, we have optimized the RNA preparatory steps and used an amplification and labeling system specifically developed for analysis of degraded RNA, along with a microarray containing probes detecting sites across the whole transcript. The genes we thereby could detect as enriched in the stroma compartment could to a large extent be identified as typical for immune cells, such as the Ig genes, and extracellular matrix, conceivably emanating from fibroblasts. This indicates that the method, despite the limited quality and quantity of the material, is able to capture compartment-specific expression patterns.

We obtained a set of 107 genes expressed at substantially higher levels in stroma. By expanding this set using correlation analysis of TCGA tumor data and successive iterations, we could define 16 gene signatures with high intra-set correlation. All of the genes identified in the expansion were also found in our data to be expressed at higher levels in the stromal compartments, supporting that they are all related to stroma.

The gene signatures conceivably represent a combination of different stromal cell types or different maturation stages of the same cell type. Sets 1 and 2 mainly contain genes typical of extracellular matrix such as *DCN*, *LUM*, *VCAN*, and collagens. These genes have been seen to be highly expressed in stroma in other studies [10,11,34,35]. We also find *FAP*, a typical myofibroblast marker, as well as *POSTN*, which is synthesized by fibroblasts in set 1. It is likely that sets 1 and 2 could be used as indicators of the amount of fibroblasts and stroma in a tumor.

Sets 4 and 5 contain genes typical of endothelial tissue, both actual angiogenic regulators *TIE1*/*TIE2* (*TEK*), *ARHGEF15*, *ROBO4*, and *ELTD1* and other endothelial markers such as *CD34*, *CLEC14A*, and *ESAM*. Several (7/12) of the genes in gene sets 4 and 5 were identified in the angiogenesis signature obtained from 1,250 tumors from different cancer types [36].

The remaining gene sets contain genes typical of immune and inflammatory cells, such as T cell-associated *CD3* (*CD3G*, *CD3D* and *CD3E*), *CD4*, *ZAP70*, *GIMAP* and granzymes (*GZMA*/*GZMK* and *GZMM*), major histocompatibility complex (MHC) II-encoding *HLA*s, and more general lymphocyte-associated genes such as *LAIR1* and *LST1*.

Cox proportional hazard analyses revealed only limited value of the gene signatures in univariate models, which suggest that neither the amount of stroma nor the extent of vascularization or the magnitude of inflammatory components has prognostic information as isolated variables. However, in a multivariate model, four of the gene signatures were significant. Sets 1 and 13 are associated with an increased risk for new tumor event, whereas gene sets 5 and 12 negatively influenced the HR.

A typical fibroblast marker (*FAP*) was identified in gene set 1 together with fibroblast-expressed extracellular matrix-associated genes, such as collagens, whereas gene set 13 contained complement factor *C1Q*. FAP has been identified as a prognostic marker in various cancer studies, including breast and lung cancer, and has been suggested to be a potential target in solid tumors [37-40]. Complement initiator C1q proteins can be derived from various stromal cells and has, in addition to its role in immune complex recognition, been found to have a proangiogenic effect in wound healing [41,42] and to drive carcinogenesis [43]. Complement activation has, on the other hand, been considered to have tumor suppressive properties, and C1q can induce apoptosis in prostate cancer cells [44,45], indicating a complex and probably context-dependent role of C1Q in tumor development.

The gene sets with negative HR contain several MHC-II-encoding *HLA* genes (gene set 12) and angiogenesis-regulating genes, such as *TEK* and *ELTD1* (gene set 5). *ELTD1* expression has previously been shown to be associated with an improved outcome [36] which may explain why this signature negatively influences the hazard. Furthermore, higher levels of *HLA-DR* have been shown to predict better prognosis of invasive ductal carcinomas [46]. The signatures were also significant when lymph node status was included and when stratifying for molecular subtype, indicating that they are independent prognostic markers.

A more limited Cox model based on only the immune gene signatures 12 and 13, with or without lymph node status and stratification for subtype in the model, could also predict recurrence. The opposing effects by these signatures on the risk indicate that the composition of the immune response in the tumor is of importance for the progression. This conclusion is further supported by the fact that the signatures are predictive in a multivariate Cox model in several other tumor forms and other breast cancer data sets. It suggests that the importance of specific components of the immune response for progression of the disease may be a general phenomenon applicable to several cancer forms.

The prognostic signatures contain mainly *C1Q* and *HLA* genes, respectively. *C1Q* genes have been shown to be produced by a number of cells, including monocytes, macrophages, and dendritic cells [42]. However, the expression of *C1Q* has previously been reported to be highest in immature immune cells that are known to reflect a state of immune paralysis in cancer immunology [47,48]. Therefore, higher expression levels of these genes could potentially reflect a higher ratio of immature, non-functional antigen-presenting cells in the tumor. Likewise, higher expression levels of *HLA* genes may be indicative of a more active immune response. This assumption is further supported by the screen (Table 5) for immune genes that are associated with a higher risk for recurrence in a multivariate model with the HLA signature. Here, we found many genes typical for negative immune signaling. These include inhibitory co-receptors that are negative regulators of immune responses (*LAIR1*, *LILRB1*, *LILRB2*, and *LILRB4*) and macrophage genes (*CD68*, *ITGB2*, and *CD86*). On the other hand, genes that were associated with a lower risk in a multivariate model with the C1Q signature (Table 6) represented genes coding for proteins involved in a cytotoxic immune response (for example, *GZMH*, *GZMA*, *GZMK*, *CD3D*, *CD3E*, *CD3G*, *CD247* (*CD3ζ*), *CD8A*, *CD27*, *CD52*, *CD96*, *PYHIN1*, *SLAMF6*, and *IL12RB1*). The number of genes with both high correlation of their expression and similar prognostic value indicates that it may be possible to identify fairly large gene sets that could be used as markers for the profile of an intra-tumor immune response. It thereby highlights potential factors determining this profile.

The results support the idea that the molecular profile of an immune response, rather than the amount of immune or inflammatory cells, is an important prognostic marker in breast cancer and in other cancer forms. Such a hypothesis is further supported by other results, including the finding that the amount of a specific set of CD4^+^ T cells, namely follicular helper T cells, predicts breast cancer survival [49].

## Conclusions

We have developed a methodological procedure for isolation and characterization of compartment-specific genes by using LCM on FFPE triple-negative breast cancers. Through expansion of the gene list with data from TCGA of correlating genes, we could identify two immune/inflammatory signatures with prognostic information. The results further underscore the importance of the composition rather than the extent of the immune response as a prognostic indicator in cancer. It also provides novel signatures that are stable indicators of prognosis and valid for some other cancer types as well and highlights genes that may be of particular importance when analyzing the composition of an immune response in a tumor.

## Additional files

Additional file 1: Table S1. Basic patient and tumor characteristics of the breast cancer. The Cancer Genome Atlas (TCGA) cohort used for the follow-up analyses is shown. Additional file 2: Table S2. RNA integrity and concentrations after membrane mounting. Additional file 3: Table S3. RNA integrity after cresyl violet staining. Additional file 4: Figure S1. RNA integrity is maintained after cresyl violet staining. Electropherogram profiles of RNA from total non-mounted tissue **(A)**, cresyl violet-stained mounted tissue with **(B)** and without **(C)** aqueous rinsing steps are shown. RNA length is indicated on the x-axis, and the y-axis corresponds to the fluorescence units. RNA integrity number (RIN) values are calculated according to a standardized algorithm by using various features correlated to RNA integrity. Additional file 5: Table S4. Genes enriched in stroma compartment, identified using the limma package in R. The magnitude of enrichment is indicated by the difference in log2 expression (logFC). Additional file 6: Table S5. Genes enriched in cancer cell compartment, identified by using the limma package in R. The magnitude of enrichment is indicated by the difference in log2 expression (logFC). Additional file 7: Figure S2. Identification of compartment-enriched genes and stroma gene sets. With the lmfit function in the limma package of R, probes that were enriched in either compartment were identified with an adjusted *P* value of less than 0.05 as cutoff. The scatter plots **(A, B)** show expression values for all probes of genes for which one probe was enriched in stroma (red) or cancer cell (blue) compartment. The black circles in (A) indicate genes for which no probe was significantly enriched in either compartment. **(C)** The expanded gene set (361 genes) are shown in red (original genes enriched in stroma) and blue (genes found after expansion with The Cancer Genome Atlas (TCGA) data). The multiplication sign denotes the genes in the final signatures. Additional file 8: Figure S3. Expression levels of gene signatures in breast cancer tumors. The mean of the log2 expression levels of the genes in each signature was calculated for 982 breast cancers using data from The Cancer Genome Atlas (TCGA) project. The aggregated values of each signature are shown for the indicated breast cancer subgroups.

## Abbreviations

DEPC: diethylpyrocarbonate
FFPE: formalin-fixed paraffin-embedded
HR: hazard ratio
LCM: laser capture microdissection
MHC: major histocompatibility complex
NHG: Nottingham histological grade
PET: polyethylene terephtalate
RIN: RNA integrity number
TCGA: The Cancer Genome Atlas
