# Supplementary material for: Prognostic stromal gene signatures in breast cancer
Source: Breast Cancer Res. 2015 Feb 21;17(1):23. doi: 10.1186/s13058-015-0530-2 (PMC4360948; doi:10.1186/s13058-015-0530-2)
Supplement: Additional file 1: Table S1. — Basic patient and tumor characteristics of the breast cancer. The Cancer Genome Atlas (TCGA) cohort used for the follow-up analyses is shown. [file 13058_2015_530_MOESM1_ESM.pdf]

Supplementary Table S1. Basic patient and tumor characteristics of the breast cancer TCGA cohort used for the analyses.

**Age**	**Median**	**Range**			
	58	26-90			
**Menopause**	**Pre-**	**Post-**	**Peri-**	**Missing**	
	117	337	14	36	
**Stage**	**Stage 1**	**Stage 2**	**Stage 3**	**Stage 4**	**Missing**
	88	299	98	6	13
**Tumor stage**	**T1**	**T2**	**T3**	**T4**	**Tx**
	140	293	56	12	3
**Node stage**	**N0**	**N1**	**N2**	**N3**	**Missing**
	252	144	51	27	30
**Metastasis stage**	**M0**	**M1**	**Mx**	**Missing**	
	443	6	55	2	
	**Positive**		**Negative**		**Missing**
**Estrogen receptor**	362		120		22
**Progesteron receptor**	315		164		25
**HER2 (immuno)**	50		254		200
**HER2 (FISH)**	21		151		332
