# Supplementary material for: Prognostic stromal gene signatures in breast cancer
Source: Breast Cancer Res. 2015 Feb 21;17(1):23. doi: 10.1186/s13058-015-0530-2 (PMC4360948; doi:10.1186/s13058-015-0530-2)
Supplement: Additional file 2: Table S2. — RNA integrity and concentrations after membrane mounting. [file 13058_2015_530_MOESM2_ESM.pdf]

Supplementary Table S2. RNA intergrity and concentrations after membrane mounting

	Extracted RNA			
	Bioanalyzer		Nanodrop	
	**(ng/µl)**	**RIN**	**(ng/µl)**	**260/280**
Tissue mounted with 30% ethanol	142	3.7	161	2.10
Non-mounted tissue	270	3.0	259	2.09
