# Supplementary material for: Prognostic stromal gene signatures in breast cancer
Source: Breast Cancer Res. 2015 Feb 21;17(1):23. doi: 10.1186/s13058-015-0530-2 (PMC4360948; doi:10.1186/s13058-015-0530-2)
Supplement: Additional file 3: Table S3. — RNA integrity after cresyl violet staining. [file 13058_2015_530_MOESM3_ESM.pdf]

Supplementary Table S3. RNA integrity after cresyl violet staining

	Extracted RNA			
	Bioanalyzer		Nanodrop	
Staining	**(ng/µl)**	**RIN**	**(ng/µl)**	**260/280**
Unstained	270	3.0	259	2.09
Cresyl violet	142	3.7	161	2.10
Cresyl violet + DEPC	59.0	4.2	73.0	2.14
