# Supplementary material for: Prognostic stromal gene signatures in breast cancer
Source: Breast Cancer Res. 2015 Feb 21;17(1):23. doi: 10.1186/s13058-015-0530-2 (PMC4360948; doi:10.1186/s13058-015-0530-2)
Supplement: Additional file 4: Figure S1. — RNA integrity is maintained after cresyl violet staining. Electropherogram profiles of RNA from total non-mounted tissue (A), cresyl violet-stained mounted tissue with (B) and without (C) aqueous rinsing steps are shown. RNA length is indicated on the x-axis, and the y-axis corresponds to the fluorescence units. RNA integrity number (RIN) values are calculated according to a standardized algorithm by using various features correlated to RNA integrity. [file 13058_2015_530_MOESM4_ESM.pdf]

Supplementary Figure S1.

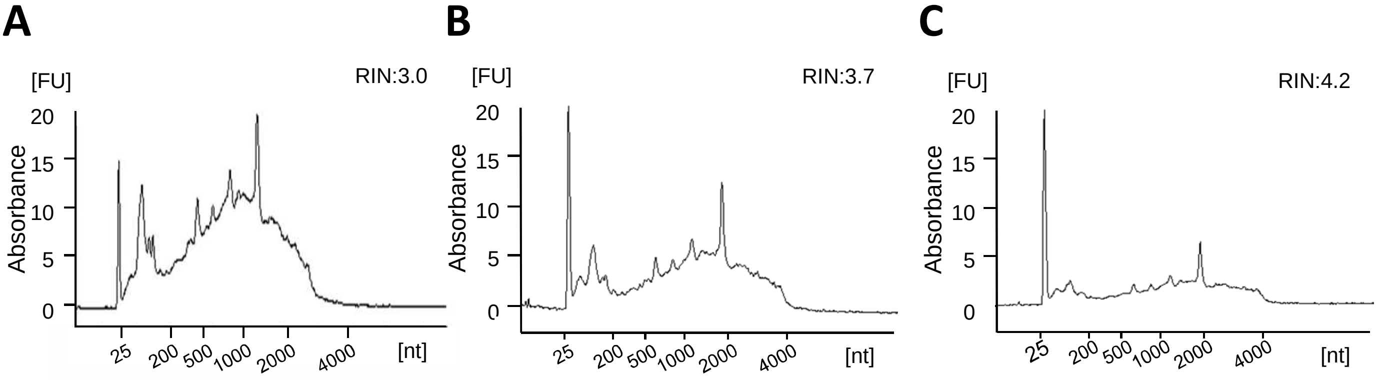

RNA integrity is maintained after cresyl violet staining. Electropherogram profiles of RNA from total non-mounted tissue (A), cresyl violet-stained mounted tissue with (B) and without aqueous rinsing steps (C). RNA length is indicated on the x-axis and y-axis corresponds to the fluorescence units. RIN values are calculated according to a standardized algorithm using various features correlated to RNA integrity.
