# Supplementary material for: Prognostic stromal gene signatures in breast cancer
Source: Breast Cancer Res. 2015 Feb 21;17(1):23. doi: 10.1186/s13058-015-0530-2 (PMC4360948; doi:10.1186/s13058-015-0530-2)
Supplement: Additional file 5: Table S4. — Genes enriched in stroma compartment, identified using the limma package in R. The magnitude of enrichment is indicated by the difference in log2 expression (logFC). [file 13058_2015_530_MOESM5_ESM.pdf]

Supplementary Table S4. Genes enriched in stroma compartment, identified using the limma package in R. The magnitude of enrichment is indicated by the difference in log2 expression (logFC).

	Symbol	logFC	adj.P.value		Symbol	logFC	adj.P.value		Symbol	logFC	adj.P.value
**1**	IGJ	-4.93	0.0036	**37**	SULF1	-2.82	0.0136	**73**	CD248	-2.35	0.0344
**2**	DCN	-4.80	0.0036	**38**	TIMP1	-2.38	0.0136	**74**	MFAP5	-2.77	0.0345
**3**	CCL19	-4.08	0.0059	**39**	BGN	-2.71	0.0141	**75**	GIMAP5	-2.08	0.0377
**4**	IL7R	-3.71	0.0066	**40**	SPARCL1	-3.17	0.0147	**76**	PDGFRB	-1.93	0.0400
**5**	LUM	-4.71	0.0066	**41**	IGHA2	-2.58	0.0155	**77**	CRISPLD2	-2.51	0.0410
**6**	MMP2	-3.71	0.0066	**42**	MXRA5	-2.63	0.0155	**78**	PIM2	-1.76	0.0425
**7**	COL1A1	-3.54	0.0079	**43**	IGK@	-3.68	0.0164	**79**	CD28	-2.05	0.0425
**8**	COL6A3	-3.59	0.0079	**44**	CILP	-2.51	0.0173	**80**	COL5A2	-2.07	0.0427
**9**	SFRP2	-4.18	0.0079	**45**	GPR183	-2.26	0.0179	**81**	CCR7	-2.06	0.0431
**10**	IGLJ3	-3.59	0.0080	**46**	SERPINE1	-2.82	0.0184	**82**	IGF2	-3.20	0.0439
**11**	IGHA1	-3.49	0.0080	**47**	FAM46C	-2.09	0.0185	**83**	IGF1	-2.93	0.0439
**12**	CDH11	-3.12	0.0089	**48**	THY1	-3.00	0.0185	**84**	CXCR4	-2.12	0.0442
**13**	IGLV6-57	-2.91	0.0089	**49**	IGFBP4	-2.43	0.0187	**85**	PRRX1	-2.14	0.0443
**14**	IGKV1-5	-3.59	0.0094	**50**	IGK@	-2.91	0.0187	**86**	LRP1	-2.54	0.0469
**15**	VCAN	-3.21	0.0096	**51**	COL12A1	-2.84	0.0215	**87**	IGK@	-3.19	0.0477
**16**	COL1A2	-3.66	0.0101	**52**	PECAM1	-2.48	0.0215	**88**	LTBP2	-1.79	0.0479
**17**	LOC652493	-2.95	0.0101	**53**	SERPINF1	-2.98	0.0222	**89**	SPARC	-2.66	0.0482
**18**	DPT	-2.94	0.0117	**54**	APOD	-2.39	0.0222	**90**	F2R	-1.79	0.0482
**19**	CTSK	-3.26	0.0124	**55**	PCOLCE	-2.33	0.0236	**91**	TAGLN	-2.31	0.0482
**20**	POSTN	-3.41	0.0124	**56**	IGKC	-2.83	0.0237	**92**	MRC1	-2.43	0.0482
**21**	FBN1	-3.15	0.0124	**57**	IGHA1	-2.77	0.0237	**93**	MRC1	-2.43	0.0482
**22**	IGKC	-2.87	0.0124	**58**	F13A1	-3.19	0.0237	**94**	FAP	-2.11	0.0482
**23**	ENPP2	-2.39	0.0124	**59**	SELPLG	-2.17	0.0246	**95**	FYN	-2.18	0.0484
**24**	IGHV4-59	-4.28	0.0124	**60**	ACTA2	-2.92	0.0255	**96**	TCF4	-2.18	0.0489
**25**	LEF1	-2.29	0.0124	**61**	PRDM1	-2.60	0.0255	**97**	SELL	-2.41	0.0489
**26**	AEBP1	-2.71	0.0124	**62**	IGHM	-3.49	0.0283	**98**	THBS2	-2.25	0.0489
**27**	CCL18	-2.99	0.0124	**63**	LAMA4	-2.29	0.0295	**99**	SLCO2B1	-2.15	0.0489
**28**	CXCL12	-3.15	0.0128	**64**	CORO1A	-2.08	0.0295	**100**	TSC22D3	-2.11	0.0489
**29**	SFRP4	-3.59	0.0133	**65**	GZMK	-2.99	0.0300	**101**	FLI1	-1.84	0.0489
**30**	TIMP3	-3.09	0.0136	**66**	CYTIP	-2.86	0.0300	**102**	DUSP1	-2.22	0.0493
**31**	CCDC80	-3.65	0.0136	**67**	CFH	-2.36	0.0305	**103**	CD4	-1.96	0.0494
**32**	SLC40A1	-2.46	0.0136	**68**	PLAC8	-2.04	0.0307	**104**	S1PR1	-1.54	0.0494
**33**	COL3A1	-3.01	0.0136	**69**	IGKC	-2.96	0.0307	**105**	ITK	-2.05	0.0494
**34**	IGHD	-4.16	0.0136	**70**	WIPF1	-2.20	0.0328	**106**	CD37	-2.11	0.0494
**35**	RARRES2	-2.23	0.0136	**71**	TIMP3	-2.23	0.0332	**107**	MMP9	-1.86	0.0494
**36**	PDK4	-3.61	0.0136	**72**	OLFML2B	-2.18	0.0336				
