# Supplementary material for: Prognostic stromal gene signatures in breast cancer
Source: Breast Cancer Res. 2015 Feb 21;17(1):23. doi: 10.1186/s13058-015-0530-2 (PMC4360948; doi:10.1186/s13058-015-0530-2)
Supplement: Additional file 6: Table S5. — Genes enriched in cancer cell compartment, identified by using the limma package in R. The magnitude of enrichment is indicated by the difference in log2 expression (logFC). [file 13058_2015_530_MOESM6_ESM.pdf]

Supplementary Table S5. Genes enriched in cancer cell compartment, identified using the limma package in R. The magnitude of enrichment is indicated by the the difference in log2 expression (logFC).

	Symbol	logFC	adj.P.value
1	DSG2	2.45	0.0124
2	RAB25	2.36	0.0136
3	MAL2	2.21	0.0136
4	ELF5	2.26	0.0136
5	VTCN1	2.34	0.0187
6	DSP	2.15	0.0215
7	IRF6	1.95	0.0215
8	LOC151009	1.98	0.0219
9	PEG10	1.93	0.0237
10	CD24	2.12	0.0246
11	PROM1	2.73	0.0280
12	ITGB8	2.39	0.0288
13	EHF	2.28	0.0295
14	KRT7	1.93	0.0295
15	MUC5B	1.92	0.0300
16	GLYATL2	2.27	0.0303
17	ELF3	1.78	0.0307
18	MPPED2	2.21	0.0307
19	MUC16	2.03	0.0327
20	BAIAP2L1	1.77	0.0328
21	PFN2	1.77	0.0344
22	ANLN	1.85	0.0344
23	DSC2	1.67	0.0377
24	TM4SF1	1.96	0.0390
25	FRMD3	1.79	0.0390
26	SORBS2	1.94	0.0409
27	ESRP1	2.50	0.0410
28	PHGDH	1.70	0.0414
29	PRSS8	2.10	0.0425
30	MYO5B	1.87	0.0443
31	TRPS1	1.94	0.0456
32	SERTAD4	1.84	0.0462
33	CLDN7	1.61	0.0474
34	AP1M2	1.71	0.0477
35	MOCOS	1.61	0.0477
36	CLDN4	1.73	0.0477
37	CKS1B	1.81	0.0484
38	CKS1B	1.77	0.0489
39	SERPINA3	1.78	0.0493
40	HIST1H2BK	1.67	0.0493
41	MYO6	1.74	0.0493
42	CHPT1	1.61	0.0494
43	UBE2T	1.60	0.0494
44	SLC7A5	1.55	0.0494
45	PTPLB	1.69	0.0494
46	GABRP	2.15	0.0494
47	GPR56	1.73	0.0499
48	MPZL2	1.95	0.0499
