# Supplementary material for: Prognostic stromal gene signatures in breast cancer
Source: Breast Cancer Res. 2015 Feb 21;17(1):23. doi: 10.1186/s13058-015-0530-2 (PMC4360948; doi:10.1186/s13058-015-0530-2)
Supplement: Additional file 7: Figure S2. — Identification of compartment-enriched genes and stroma gene sets. With the lmfit function in the limma package of R, probes that were enriched in either compartment were identified with an adjusted P value of less than 0.05 as cutoff. The scatter plots (A, B) show expression values for all probes of genes for which one probe was enriched in stroma (red) or cancer cell (blue) compartment. The black circles in (A) indicate genes for which no probe was significantly enriched in either compartment. (C) The expanded gene set (361 genes) are shown in red (original genes enriched in stroma) and blue (genes found after expansion with The Cancer Genome Atlas (TCGA) data). The multiplication sign denotes the genes in the final signatures. [file 13058_2015_530_MOESM7_ESM.pdf]

## Supplementary Figure S2.

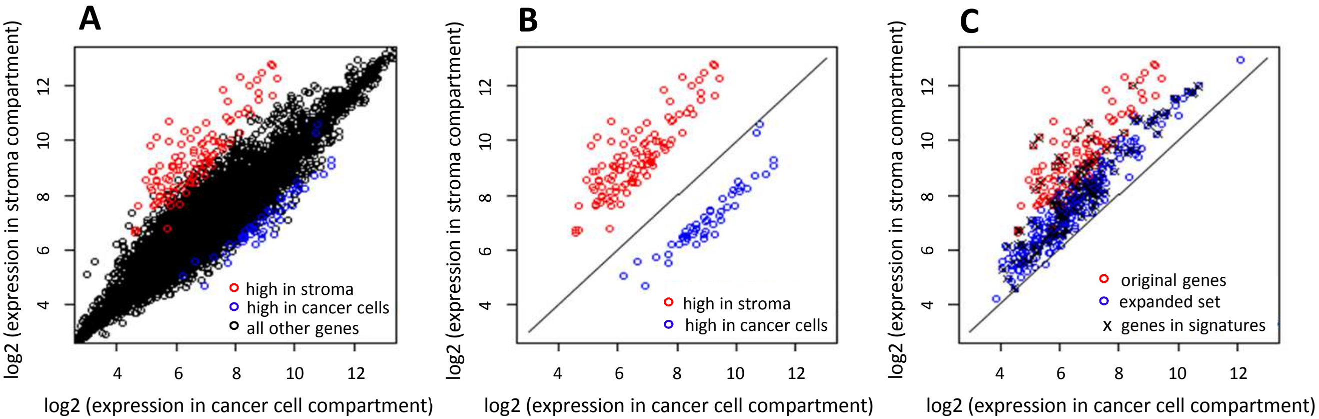


**Identification of compartment-enriched genes and stroma gene sets.** Using the lmfit function in the limma package of R, probes that were enriched in either compartment were identified with an adjusted p-value<0.05 as cutoff. The scatter plots (A and B) show expression values for all probes of genes for which one probe was enriched in stroma (red) or cancer cell (blue) compartment. The black circles in (A) indicate genes for which no probe was significantly enriched in either compartment. In (C) the expanded gene set (361 genes) are shown in red (original genes enriched in stroma) and blue (genes found after expansion with TCGA data); × denotes the genes in the final signatures.
