# Supplementary material for: Prognostic stromal gene signatures in breast cancer
Source: Breast Cancer Res. 2015 Feb 21;17(1):23. doi: 10.1186/s13058-015-0530-2 (PMC4360948; doi:10.1186/s13058-015-0530-2)
Supplement: Additional file 8: Figure S3. — Expression levels of gene signatures in breast cancer tumors. The mean of the log2 expression levels of the genes in each signature was calculated for 982 breast cancers using data from The Cancer Genome Atlas (TCGA) project. The aggregated values of each signature are shown for the indicated breast cancer subgroups. [file 13058_2015_530_MOESM8_ESM.pdf]

Supplementary Figure S3.

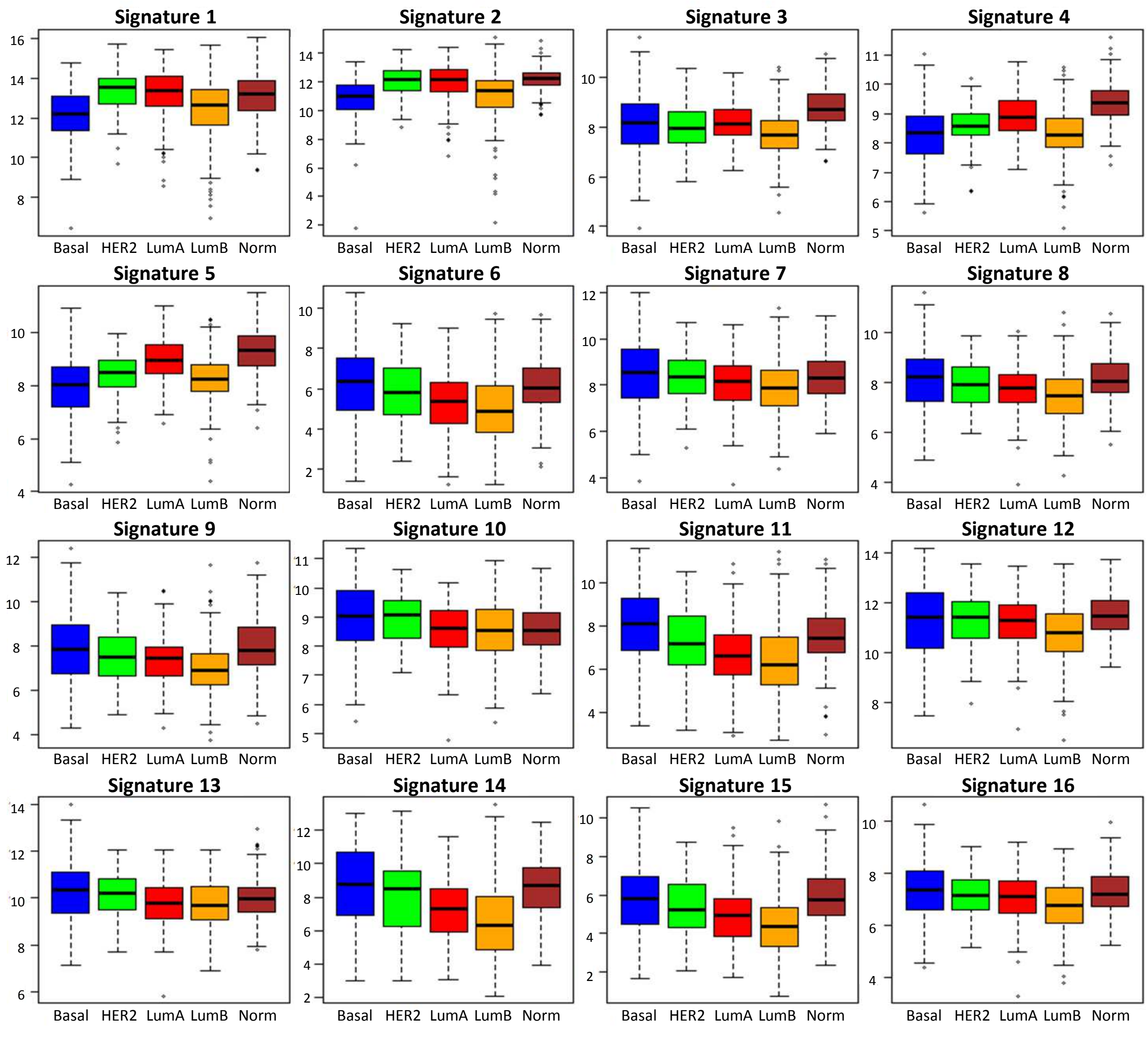

Expression levels of gene signatures in breast cancer tumors. The mean of the log2 expression levels of the genes in each signature were calculated for 982 breast cancers using data from the TCGA project. The aggregated values of each signature is shown for the indicated breast cancer subgroups.
